# Wet-Chemical Synthesis and Applications of Semiconductor Nanomaterial-Based Epitaxial Heterostructures

**DOI:** 10.1007/s40820-019-0317-6

**Published:** 2019-10-15

**Authors:** Junze Chen, Qinglang Ma, Xue-Jun Wu, Liuxiao Li, Jiawei Liu, Hua Zhang

**Affiliations:** 1 Center for Programmable Materials, School of Materials Science and Engineering, Nanyang Technological University, 50 Nanyang Avenue, Singapore, 639798 Singapore; 2 State Key Laboratory of Coordination Chemistry, School of Chemistry and Chemical Engineering, Nanjing University, Nanjing, 210023 People’s Republic of China; 3 Department of Chemistry, City University of Hong Kong, Kowloon, Hong Kong People’s Republic of China

**Keywords:** Heterostructure, Nanoarchitecture, Epitaxy, Wet-chemical synthesis, Semiconductor nanomaterial

## Abstract

Semiconductor nanomaterial-based epitaxial heterostructures with precisely controlled compositions and morphologies are of great importance for various applications in optoelectronics, thermoelectrics, and catalysis. Until now, various kinds of epitaxial heterostructures have been constructed. In this minireview, we will first introduce the synthesis of semiconductor nanomaterial-based epitaxial heterostructures by wet-chemical methods. Various architectures based on different kinds of seeds or templates are illustrated, and their growth mechanisms are discussed in detail. Then, the applications of epitaxial heterostructures in optoelectronics, catalysis, and thermoelectrics are described. Finally, we provide some challenges and personal perspectives for the future research directions of semiconductor nanomaterial-based epitaxial heterostructures.

## Highlights

- The synthesis of semiconductor nanomaterial-based epitaxial heterostructures by wet-chemical methods is introduced. Various architectures based on different kinds of seeds or templates are illustrated, and their growth mechanisms are discussed in detail.
- The applications of epitaxial heterostructures in optoelectronics, thermoelectrics, and catalysis are discussed.

## Graphical abstract

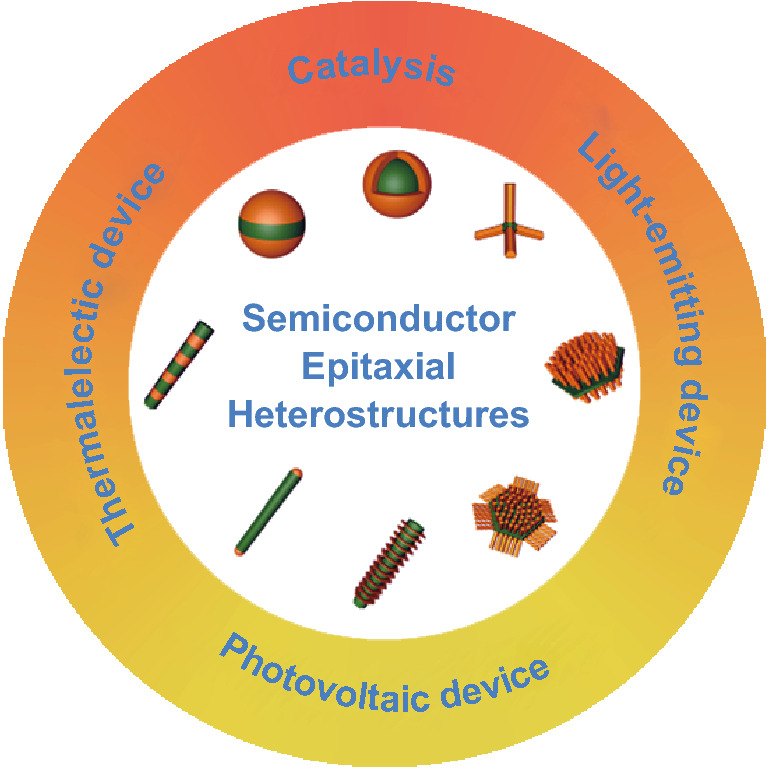

## Introduction

The construction of semiconductor nanomaterial-based heterostructures with tunable compositions and morphologies is essential for various applications in electronics, optoelectronics, thermoelectrics, catalysis, etc. [1–10]. The interface between different components of these heterostructures plays a critical role in determining the performance of devices. Unintentional interfacial defects, such as polycrystallinity and dislocation, lead to the performance degradation and even premature failure of devices. For example, interfacial defects generate lots of problems in metal oxide semiconductor devices, including the frequency dispersion of capacitance and suboptimal electron mobility [11, 12]. Therefore, to achieve high-quality semiconductor interfaces is essential for the fabrication of high-quality devices.

Epitaxial growth, by which the atoms in a thin crystalline layer grown onto another single-crystalline substrate mimic the atomic arrangement of the substrate, enables the creation of high-quality interface [13–15]. An epitaxial interface with few interfacial defects increases the electron mobility across the junction, thus improving the device performance. The successful attempt of epitaxy dates back to 1836, when Frankenheim demonstrated a parallel-oriented growth of sodium nitrate on calcium carbonate [16]. This research area was promoted by the emergence of semiconductor industry in the early 1960s. In the past few decades, with the development of nanotechnology, the construction of epitaxial semiconductor-based heterostructures has attracted tremendous attention due to their potential applications in electronics [17–19], optoelectronics [20, 21], thermoelectrics [22, 23], and catalysis [24]. Now, a large variety of electronic, optoelectronic, magnetic, and superconducting devices have been fabricated using epitaxial techniques.

**Fig. 1:**
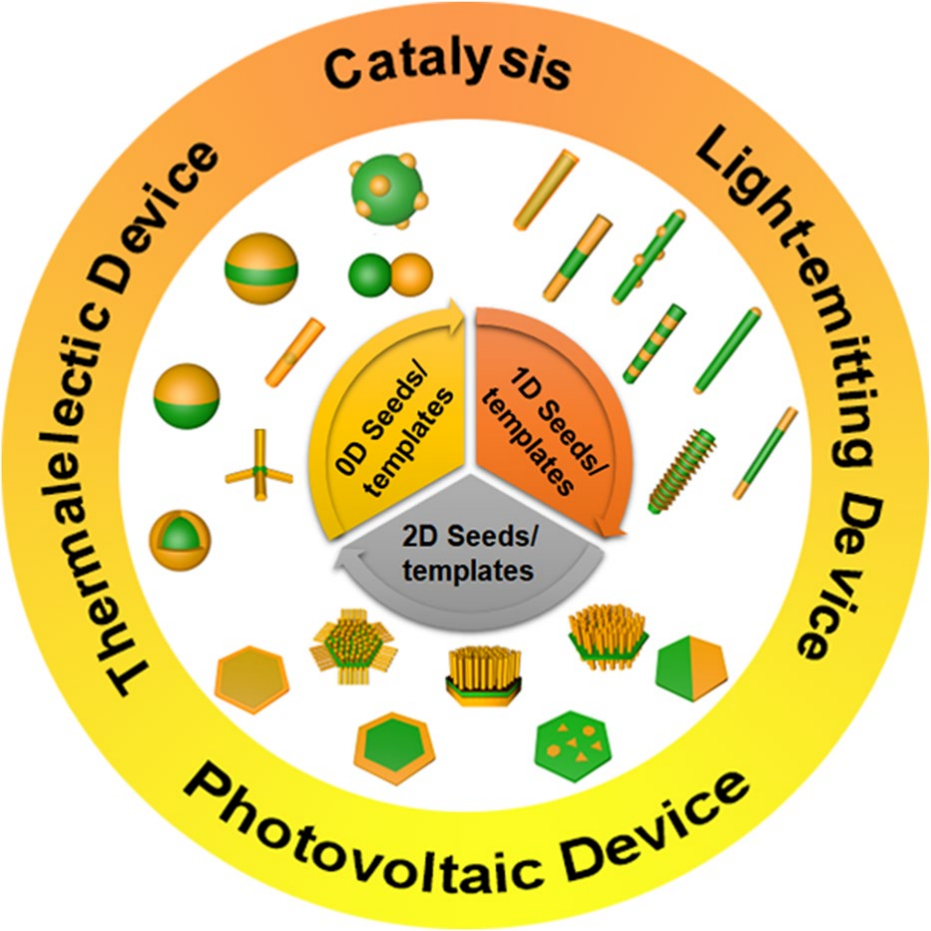
Schematic illustration of epitaxial heterostructures based on 0D seeds/templates, 1D seeds/templates, and 2D seeds/templates

The construction of epitaxial semiconductor heterostructures can be realized by several approaches, including liquid-phase epitaxy, metal organic vapor-phase epitaxy, and molecular beam epitaxy [25–29]. Although high-quality epitaxial heterostructures have been obtained, the development of the fabrication techniques is impeded by the low yield, high cost, and stringent reaction conditions, such as high temperature and ultra-high vacuum. In contrast, wet-chemical synthetic methods are attractive alternatives due to the low-cost and high-yield production. More importantly, they offer more opportunities for engineering the architectures of heterostructures. Until now, various kinds of semiconductor-based epitaxial heterostructures with modulated compositions and fine-tuned morphologies have been synthesized by using zero-dimensional (0D), one-dimensional (1D), and two-dimensional (2D) nanocrystals (NCs) as seeds or templates via wet-chemical methods (Fig. 1). For example, starting from 0D seeds, core@shell, Janus, rod-like, and tetrapod nanostructures can be obtained by changing the reaction conditions. This minireview summarizes the state-of-the-art progress on the semiconductor-based epitaxial heterostructures. A brief introduction of this field is first described, followed by the discussions on the synthetic methods and growth modes of epitaxial heterostructures. Then, we will elaborate the various architectures of epitaxial heterostructures based on different kinds of seeds, with particular focus on the growth mechanism. After that, we will discuss the unique advantages of these epitaxial heterostructures in some applications, including optoelectronics, thermoelectrics, and catalysis. Finally, based on the current research achievements, we will provide some personal insights into the challenges and the future research directions in this field.

## Synthesis Methods

Epitaxial heterostructures can be constructed using a variety of wet-chemical methods, including the direct synthesis from molecular precursors (e.g., solution epitaxial growth) and the post-synthesis treatments of the existing seeds or templates (e.g., ion exchange). In the solution of epitaxial growth methods, the nucleation of the second material is allowed on the defined sites of existing seeds. Ion exchange methods, especially the cation exchange in which the cations in a NC host lattice are substituted for those in solution, have been used as a particularly powerful tool for the construction of epitaxial heterostructures recently.

### Solution Epitaxial Growth

“Seeds” are always required for the construction of epitaxial heterostructures in solution epitaxial growth based on different synthesis strategies, e.g., hot-injection and hydro-/solvothermal strategies. The energy barrier for the homogeneous nucleation in solution is typically quite large, but it would be different if seeds present in the nucleation solution, i.e., heterogeneous nucleation. The size, shape, crystal structure, and surface property of the seeds greatly affect the nucleation and growth of the second materials in a variety of ways.

#### Epitaxial Growth Modes

Similar to the traditional thin-film epitaxy by vapor-phase techniques, there are three different growth modes for constructing epitaxial heterostructures in solution, i.e., layer-by-layer deposition (Frank–van der Merwe mode), island growth (Volmer–Weber mode), and layer-by-layer growth followed by island growth (Stranski–Krastanov mode) [30]. The aforementioned three growth modes are determined by both lattice strain and surface free energy. When a second material is deposited on an existing seed of different materials, the excess energy is calculated by Eq. 1:

1 $$\Delta \gamma = \gamma_{1} + \gamma_{i} {-}\gamma_{2}$$

where *γ*_1_ and *γ*_2_ are surface energies (i.e., the solid/solution interfacial energies) of the deposited second material and the seed, respectively, and *γ*_i_ is the solid/solid interfacial energy [31]. The surface energy is dependent on the adhesion of foreign species, such as solvents, capping ligands, and monomers. The solid/solid interfacial energy depends on the bonding strength and the crystallographic compatibility (crystal structure and lattice constant).

If the mismatch of interfacial energy between two materials is small (*γ*_i_ is small) and the deposited material exposes lower energy surface (*γ*_1_ < *γ*_2_), the deposition would likely take place in the layer-by-layer mode, resulting in a continuous and uniform coverage (∆*γ* < 0, Frank–van der Merwe). On the other hand, if the second material is featured by a higher energy surface (i.e., *γ*_1_ > *γ*_2_) and the mismatch of interfacial energy between two materials is relatively large (*γ*_i_ is large), the second material tends to grow on the high-energy site of the seed and form islands in order to minimize strain energy. This is the so-called Volmer–Weber or island growth mode (∆*γ* > 0). The third growth model, i.e., the Stranski–Krastanov or island-on-wetting-layer growth mode, happens in systems with relatively large lattice mismatch between the two materials and with a negative ∆*γ* at the initial growing stage. Therefore, in the early stage, the second material forms based on the layer-by-layer growth mode (∆*γ* < 0). The strain energy increases linearly with the deposited layers. Subsequently, as the deposited layer exceeds a critical thickness, ∆*γ* becomes positive and the growth changes from the layer-by-layer growth to the island growth on the wetting layer to release the strain energy.

#### Synthesis Strategies

##### Hot-Injection Strategy

The hot-injection strategy for the synthesis of monodisperse, highly luminescent cadmium chalcogenide NCs is a very efficient approach to prepare epitaxial heterostructures with diverse morphologies. In the hot-injection method, a “cold” (room temperature) precursor solution was rapidly injected into a hot reaction solution at high temperature, which effectively separates the nucleation and the further growth of NCs. Thus, the size distribution of the synthesized NCs is remarkably narrow. However, the thermodynamics and kinetics in the synthesis of epitaxial heterostructures are quite different with that in the synthesis of single-phase NCs. Generally, the preparation of epitaxial heterostructures is a heterogeneous nucleation process; thus, the free energy barrier is smaller than that during the preparation of single-phase NCs. To reduce the self-nucleation in solution, the reaction conditions should be carefully controlled. Taking the synthesis of CdSe@CdS core@shell epitaxial heterostructure as an example, instead of rapid injection, 1-octadecene (ODE) solution containing cadmium (II) oleate (Cd-oleate) and octanethiol was injected dropwise at a rate of 3 mL h^−1^ using a syringe pump into the growth solution containing CdSe seeds [20]. The shell thickness can be precisely controlled by adjusting the injection amount of the precursor.

A distinct advantage of the hot-injection strategy is that it allows the one-pot synthesis of complex epitaxial heterostructures through multiple injections of a sequence of precursors. Therefore, multi-shell structures and branched structures can be obtained via one-pot synthesis. In order to prepare CdTe-CdSe tetrapod heterostructures, a stock solution of trioctylphosphine telluride (TOP-Te) was first injected into the reaction solution containing Cd-oleate, resulting in the formation of CdTe seeds. After that, a trioctylphosphine-selenide (TOP-Se) stock solution was injected to initiate the growth of CdSe arms on the surface of CdTe seeds, leading to the formation of CdTe-CdSe tetrapods [32]. By carefully selecting the precursor, epitaxial heterostructures can also be synthesized by one-injection method. For example, our group realized the synthesis of CdS-MoS_2_ and CdS-WS_2_ heterostructures by injecting (NH_4_)_2_MoS_4_ or (NH_4_)_2_WS_4_ stock solution into a reaction solution containing Cd-oleate [33]. The thermal decomposition of (NH_4_)_2_MoS_4_ or (NH_4_)_2_WS_4_ not only provided the sulfur source for the nucleation and growth of CdS seeds, but also acted as the precursor which decomposed to form MoS_2_ or WS_2_ nanosheets (NSs).

##### Hydro-/Solvothermal Strategy

Epitaxial heterostructures can also be prepared by the hydro-/solvothermal strategy, a typical wet-chemical synthesis method, which uses water or organic solvents as the reaction medium in a sealed steel pressure reactor with Teflon liner. The chemical reaction can proceed at the temperature higher than the boiling point of the solvent. Under such condition, high pressure can be generated, promoting the reaction and improving the crystallinity of the synthesized NCs [34]. The main advantage of the hydro-/solvothermal strategy is that almost all precursors can dissolve in the solvent at high pressure and temperature. Besides that, the merits of hydro-/solvothermal strategy, including the easy operability, high yield, and low cost, make it an attractive choice for constructing epitaxial heterostructures. As a typical example, Wang et al. reported the epitaxial growth of α-Fe_2_O_3_ on CdS nanowires (NWs) via a two-step solvothermal process using N,N-dimethylformamide as solvent and poly(vinylpyrrolidone) as surfactant [35].

Even though hydro-/solvothermal strategy has been widely used to prepare epitaxial heterostructures, it has disadvantages as well. Firstly, it is impossible to observe the reaction process in situ since the reaction occurs in a sealed autoclave, making it difficult to propose the growth mechanism. Besides, since the hydro-/solvothermal reaction is very sensitive to the experimental condition, including the monomer concentration, surfactant, solvent, and temperature, it is difficult to precisely control the synthesized nanostructures.

### Ion Exchange

Ion exchange methods, recently, become more and more popular. In the past decades, a variety of epitaxial heterostructures have been directly synthesized from molecular precursors, which provide numerous templates for the ion exchange. These well-controlled epitaxial heterostructures can be easily transformed to other heterostructures with different compositions but similar structures with the original ones. In addition, partial ion exchange can also create epitaxial heterostructures that are difficult to be directly synthesized from molecular precursors.

#### Cation Exchange

Cation exchange, i.e., the cations in a NC are replaced with new cations, while the original anion sublattice is preserved, has been demonstrated as a very efficient chemical transformation approach to prepare a wide variety of materials and nanostructures [36–38]. The fast development of solution-phase epitaxial growth provides lots of well-controlled epitaxial heterostructures that can be used for the cation exchange. For example, the Cu_2_Se-Cu_2_S and PbSe-PbS dot-in-rod structures can be easily obtained by performing cation exchange reaction in the CdSe-CdS dot-in-rod structure [39].

Interestingly, by performing partial cation exchange, which just extracts a fraction of the original cations in the starting NCs and replaces them with new types of cations, the original single-phase nanostructure can be transformed to either alloy/doped nanostructure or various types of heterostructures. The experimental results mainly depend on the miscibility of materials before and after the cation exchange. If the reactant and product are miscible, alloy/doped nanostructures can be obtained. Otherwise, in immiscible systems, multi-domain heterostructures, such as core@shell or segmented NCs, can be obtained. In this minireview, we only focus on the immiscible system. As the rigid anion sublattice keeps unchanged, different domains possessing similar anion structure usually lead to a perfect epitaxial heterostructure. For example, core@shell structures, such as CdS@PbS [40], CdSe@PbSe [41], CdS@HgS [42], Cu_2-x_Se@SnSe [43], Cu_2-x_Se@ZnSe [43], and CdTe@Cu_2-x_Te [44], have been obtained by partial cation exchange. Besides the most common core@shell structure, lots of segmented structures that are difficult to be directly synthesized from molecular precursors, such as Janus and sandwich-like heterostructures, can be obtained by partial cation exchange. For example, Regulacio et al. reported a one-pot method to synthesize the Cu_1.94_S-CdS Janus heterostructure [45]. In their experiment, the Cu_1.94_S nanoplate with a diameter of around 40 nm was first formed. Then, the Cu_1.94_S-CdS Janus heterostructure was obtained via partial cation exchange by rapid addition of Cd precursor into the reaction solution containing Cu_1.94_S nanoplates.

#### Anion Exchange

Different from the cation exchange, which preserves the original shape of the starting template, the anion exchange is often accompanied by the nanoscale Kirkendall effect, resulting in the great change of the template morphology. The chemical transformation of NCs accompanied by the Kirkendall effect often results in the formation of polycrystalline NCs. However, in some cases, epitaxial heterostructures can also be obtained by anion exchange. For example, Park et al. demonstrated that ZnO@ZnS yolk@shell NCs were obtained by partial anion exchange using ZnO as the starting template [46]. Importantly, the crystal symmetry and orientation of the initial ZnO NC are well preserved after anion exchange, leading to the formation of ZnO@ZnS epitaxial heterostructure.

## Architectures of Semiconductor Nanomaterial-Based Epitaxial Heterostructures

To date, semiconductor nanomaterial-based epitaxial heterostructures with various architectures and compositions have been prepared via wet-chemical methods. Since the seeded growth is often involved during the synthesis processes, in this section, we will introduce different types of nanostructures based on the starting seeds/templates. Epitaxial heterostructures based on 0D, 1D, and 2D seeds/templates will be described.

### Semiconductor Nanomaterial-Based Epitaxial Heterostructures with 0D Seeds/Templates

The most common structure of nanomaterials is 0D NCs, which have been extensively studied in the past decades. However, starting from such simple configuration, various epitaxial structures, including core@shell structures, nanodimers, nanoflowers, 1D nanorods, tetrapods, and Janus nanostructures, can be obtained by using 0D NCs as seeds.

#### 0D NCs Grown on 0D Seeds

##### Core@shell Nanostructures

The core@shell configuration formed by the growth of a crystalline overlayer on 0D seed is the most common heterostructure. Originally, the construction of epitaxial core@shell structures was served to prepare core@shell quantum dots (QDs) with inorganic passivating shells to enhance the photoluminescence (PL) quantum yields. Till now, numerous epitaxial core@shell and core@multi-shell nanostructures made of group II–VI, IV–VI, and III–V semiconductors, such as CdSe@CdS [20, 47–49], CdS@ZnS [50–53], InP@ZnS [54–57], PbSe@PbS [58–61], PbSe@CdSe [62–64], ZnTe@CdSe [65–67], and CdSe@ZnTe-ZnS [68–70], have been prepared to enhance the PL, improve the stability, and tune the emission wavelength. The basic properties of different types of core@shell and core@multi-shell nanostructures with different chemical compositions and band alignment structures have been well summarized in several recent reviews [71–73]. Here, we only focus on the key factors that affect the construction of epitaxial configurations.

Similar crystal structure with small lattice mismatch between the core and shell materials is the prerequisite for the construction of high-quality epitaxial core@shell nanostructure. For example, Cao et al. examined the effect of lattice mismatch on the growth and electronic properties of InAs@shell NCs [74]. Various shell materials with different values of lattice mismatch, ranging from nearly zero for CdSe shell to as high as 10.7% for ZnS shell, were investigated. The PL quantum yield of InAs@CdSe increased up to 20% as compared to 8% for that of InAs@ZnS. This is probably due to the very large mismatch between ZnS and InAs. Cracks might be formed at the early growth stage at the interface of InAs@ZnS due to the large strain. Moreover, ZnS NCs preferentially crystallized in the wurtzite structure (the InAs core was in zinc blende structure), which may create additional defects upon the shell growth. Similar results were observed in CdSe@CdS and CdSe@ZnS systems [75].

The lattice mismatch seriously limits the possibility to grow a shell on the core with significant thickness, but without introducing defects, which are detrimental to the PL property. As the shell thickness increases, the lattice strain builds up to a point at which it is no longer to be released through the elastic crystal deformation. To remedy this drawback, core@multi-shell nanostructure is synthesized, in which the core is buried within shells made of consecutive layers of two or three semiconductors with gradually diverging lattice parameters. A lattice parameter adaptation layer sandwiched between the core NC and an outer shell allows the strain to be released gradually. Therefore, structural defects can be minimized, and the PL efficiency and stability can be greatly improved. This strategy has been used in the CdSe@ZnSe-ZnS core@shell–shell system. For ZnSe and ZnS, the lattice mismatch relative to CdSe is 7% and 10.7%, respectively. Bleuse et al. reported a CdSe@ZnSe-ZnS core@shell–shell heterostructure in which the intermediate ZnSe layer acted as a lattice parameter adaptation layer, which reduced the structural defects upon the epitaxial growth of the ZnS shell [76]. The CdSe@ZnSe-ZnS system showed higher stability in the photo-oxidation process compared to the CdS@ZnSe core@shell structure and higher photoluminescence efficiency than the CdSe@ZnS nanostructure. This strategy has also been extended to the CdSe@CdS-ZnS core@shell–shell heterostructure [77].

##### Nanodimers

The formation of core@shell structures is not favored if the lattice mismatch is too large. In this case, the nanodimer structure may be formed to minimize the interfacial energy resulting from the lattice mismatch by reducing the interfacial area between the seed and growth materials [78]. This phenomenon has been exploited in the growth of *γ*-Fe_2_O_3_-MS (M = Zn, Cd, Hg) [79] and CdS-FePt [80] nanodimers. For example, CdS-FePt nanodimers have been synthesized by injecting the sulfur precursor into a reaction solution containing Cd-oleate precursor and FePt seeds with different diameters. At the early growth stage, FePt-CdS core@shell structure with a highly defective and strained CdS shell was obtained. To release the strain, the CdS shell coalesced and formed a separated grain upon being annealed at high temperature. Using 4-nm FePt as seed, all CdS aggregated into a single grain, forming a nanodimer structure. In contrast, by using 9-nm FePt as seed, a few grains were formed because of the limited diffusion length of CdS, thus obtaining flower-like structures after annealing.

The effect of lattice mismatch on the formation of heterostructures has been studied by comparing the growth of ZnS, CdS, and HgS on *γ*-Fe_2_O_3_ NCs. All these group II–VI semiconductors possess the zinc blende structure with systematically increased lattice parameters. For ZnS, which has the smallest lattice mismatch with *γ*-Fe_2_O_3_, several domains of ZnS formed on different (111) facets of *γ*-Fe_2_O_3_ seeds (not a uniform coating as in the core@shell structure), resulting in the formation of flower-like structure. For CdS, which has a larger mismatch, the majority of the resulting *γ*-Fe_2_O_3_-CdS heterostructures are nanodimers, since the growth of CdS on *γ*-Fe_2_O_3_ is less favorable. For HgS, which has the largest lattice mismatch, only a few dimers are formed with most of HgS nucleating individually in the solution, forming isolated HgS particles.

#### 1D NCs Grown on 0D seeds

##### Dot-in-Rod

**Fig. 2:**
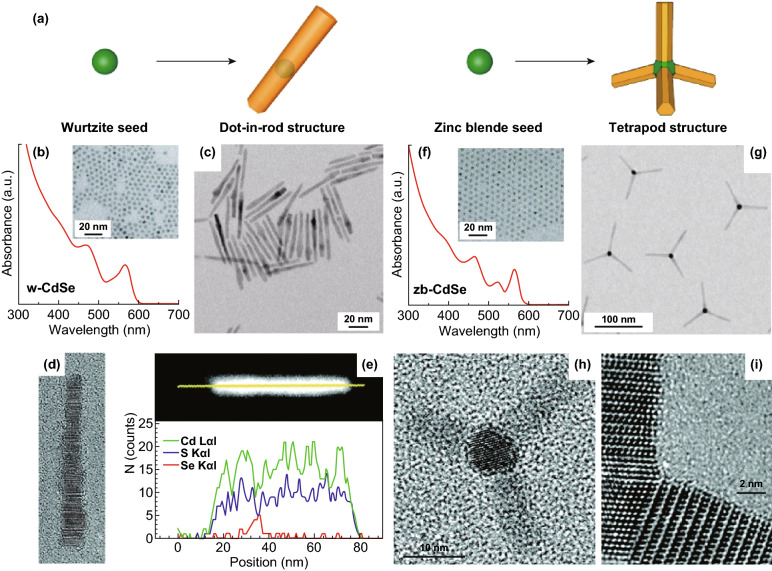
**a** Schematic illustrations of dot-in-rod and dot-in-tetrapod structures obtained from wurtzite and zinc blende seeds, respectively. **b** Absorption spectrum and TEM image of 4.4-nm CdSe NCs with wurtzite lattice. **c** TEM image of CdSe-CdS dot-in-nanorod structures. **d** High-resolution TEM image of a CdSe-CdS dot-in-nanorod grown from a 4.4-nm w-CdSe seed. Scale bar, 5 nm. **e** High-angle annular dark-field (HAADF) image of a CdSe-CdS dot-in-rod and corresponding elemental profiles for Cd, S, and Se obtained by recording energy dispersion spectroscopy signal intensities along the line shown in yellow in HAADF image. **f** Absorption spectrum and TEM image of 4.0-nm CdSe nanocrystals with zinc blende lattice. **g** TEM images of CdSe/CdS tetrapods grown from 4.0-nm zb-CdSe seeds. **h** TEM image of a CdSe/CdS tetrapod grown from 4-nm zb-CdSe seed. **i** HRTEM image of a tetrapod fragment showing the interface between {111} planes of zb-CdSe seed and {001} planes of w-CdS arms. Reproduced with permission from Ref. [82]. Copyright 2007, American Chemical Society

Besides the lattice mismatch, the crystal structure of the seed and the capping molecule also affect the final morphology of the obtained heterostructure. Asymmetric growth may be initiated by manipulating the reaction kinetics. For example, Talapin et al. reported the semiconductor heterostructure with mixed dimensionality composed of 0D CdSe core and 1D rod-like CdS shell [81, 82]. The CdSe-CdS dot-in-rod structure was synthesized by slow addition of Cd and S precursors into a reaction solution containing the wurtzite CdSe seed at a temperature of 130 °C (Fig. 2a–e). The asymmetric growth could be understood based on the intrinsic difference of the facets of wurtzite NCs, in which the NCs were terminated mainly by {100}, {001}, and {00 $\bar{1}$ } facets. Two factors led to the asymmetric growth. First, the lattice mismatch between wurtzite CdSe (w-CdSe) and CdS was larger along the < 001 > direction (~ 4.2%) than that along the perpendicular < 100 > direction (~ 3.8%). Thus, the epitaxial growth was favorable on the {001} and {00 $\bar{1}$ } facets due to the small interfacial strain. Second, Cd atoms on {00 $\bar{1}$ } facet had more dangling bonds than those on the other two facets, making the {00 $\bar{1}$ } facet most active. The reaction kinetics was governed by the concentration of the precursors, reaction temperature, and surfactant. At a low reaction temperature (i.e., 130 °C), the onion-like CdSe@CdS core@shell structure was formed when the molar ratio of Cd and S precursors was in the range of 1:1 to 1:1.6. When excess S was used (i.e., Cd/S = 1:3 to 1:5), asymmetric growth of the CdS shell was initiated and the dot-in-rod structure was obtained. A large excess of the S precursor in the synthesis was essential for the asymmetric growth, since the S interacted preferentially with the active {00 $\bar{1}$ } facet, promoting the asymmetric nucleation of CdS on the surface of CdSe core. Moreover, the presence of hexadecylamine surfactant further promoted the growth of CdS along the *c*-axis. However, at a high reaction temperature (i.e., 280 °C), the core@shell structure was obtained despite the molar ratio of the precursors, because the energy difference among different facets became negligible at high temperature, resulting in a transition from asymmetric to symmetric growth of the CdS shell.

##### Branched Structures

Since the asymmetric growth is realized by selective nucleation of CdS nanorods on different facets of CdSe seeds, it is possible to engineer the shape of heterostructures by tailoring the crystal structure of the seeds. For example, by using CdSe NCs with wurtzite (w-CdSe) or zinc blende (z-CdSe) structure as seeds for the growth of CdS nanorods, the CdSe-CdS dot-in-rod structure or tetrapods were obtained, respectively (Fig. 2a) [82, 83]. The zinc blende CdSe has four {111} facets that were analogous to the {00 $\bar{1}$ } facet of wurtzite structure, which are more favorable for the epitaxial growth of CdS nanorods. Thus, the tetrapods with four CdS “arms” grown on the surface of CdSe NCs were realized when the zinc blend CdSe NCs were used as seeds (Fig. 2f–i) [82]. This method has been extended to prepare ZnTe-CdSe dot-in-rod/tetrapods [84] and CdSe-CdTe tetrapods [85]. More recently, it became apparent that the size of the seed also plays a crucial role in determining the final morphology of the synthesized heterostructures. CdTe-CdSe heterostructures with a variety of shapes, including nanorod, Y-shaped structure, and tetrapod, can be prepared by simply changing the size of zinc blende CdTe seeds from 2.6, 4.3 to 8.2 nm, respectively [86]. Interestingly, octapod structures made by CdX (*X* = S or Se) nanorods grown on CdSe cores were obtained by using cubic Cu_2-*x*_Se NCs with the size of around 10–15 nm as seeds [87]. Due to the fast cation exchange of Cu_2-*x*_Se in the presence of excessive Cd^2+^ ions, there is no Cu_2-*x*_Se observed in the obtained octapod structure. Instead, branched CdSe-CdX NCs were obtained which composed of a cubic CdSe core and wurtzite CdX arms. In addition, epitaxial branched structures made of metal core and semiconductor arms have also been prepared [88]. Because of the relatively weak interaction between many semiconductors and metals and their large lattice mismatch, the construction of branched structures made of metal cores is more difficult compared with the synthesis of branched structures with semiconductor seeds.

#### Other Structures Based on 0D Seeds

**Fig. 3:**
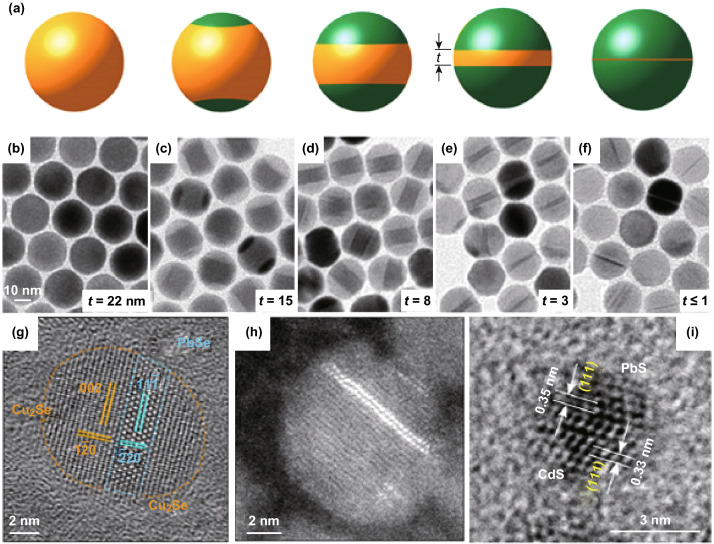
**a** Schematic illustration of the cation exchange transformation of Cu_1.8_S NCs into sandwich-like heterostructures with ZnS caps. **b–f** TEM images show the composition change from the initial Cu_1.8_S NCs **b** into ZnS **f** with a thin layer of Cu_1.8_S. The Cu_1.8_S disk thickness (t) is indicated. Reproduced with permission from Ref. [89]. Copyright 2014, American Chemical Society. **g** Scanning transmission electron microscopy (STEM) image of a single partially exchanged hexagonal Cu_2_Se NC. Two PbSe stripes in the middle of the NC were observed. Reproduced with permission from Ref. [90]. Copyright 2017, American Chemical Society. **h** TEM image of partially exchanged CdS QD consisting of a heterostructure of CdS and PbS. **i** TEM image of partially exchanged CdSe QD. Reproduced with permission from Ref. [91]. Copyright 2015, American Chemical Society

Novel and complex heterostructures, such as sandwich-like and Janus structures, are difficult to be directly synthesized from molecular precursors, but they can be easily obtained through the partial cation exchange method. For example, Ha et al. reported the synthesis of dual-interface Cu_2-*x*_S/ZnS heterostructures consisting of disk-like Cu_2-*x*_S layer sandwiched between two ZnS caps (Fig. 3a) [89]. During the cation exchange process, ZnS grains nucleated symmetrically on the two opposite sides of Cu_2-*x*_S NCs and the Cu_2-*x*_S in the center became a disk-like 2D layer as the ZnS grains grew. The thickness of Cu_2-*x*_S can be well tuned by controlling the extent of cation exchange (Fig. 3b–f). Surprisingly, an atomic thin layer of 2D Cu_2-*x*_S within a ZnS NC with epitaxial connection can be obtained, providing a template for investigating some unique physical properties in NCs, such as 2D hole gas and 2D quantum well. Sandwich-like heterostructures can also be obtained when the cation exchange starts at the middle of NCs. For example, Gariano et al. demonstrated that in hexagonal Cu_2_Se NCs, the cation exchange of Cu^+^ with Pb^2+^ generated the PbSe stripe sandwiched between hexagonal Cu_2_Se domains (Fig. 3g, h) [90]. The sandwiched structure was formed as a result of the preferential diffusion of Pb^2+^ ions through the specific (*a,b*) planes of hexagonal Cu_2_Se NCs.

Cation exchange can also start from one side of a NC, resulting in the formation of Janus heterostructure. Zhang et al. reported the preparation of Janus-like CdX/PbX (X = S or Se) heterostructures by partially exchanging the Cd^2+^ cation with the Pb^2+^ cation [91]. The cation exchange started anisotropically from one side of the original CdX NC and proceeded along the < 111 > direction, resulting in the formation of Janus heterostructure with shaped (111)_Pb*X*_/(111)_Cd*X*_ epitaxial interface (Fig. 3i). Such configuration minimizes both the strain and interfacial energy. Theoretical calculations indicated that an interfacial Se atomic layer was sandwiched by a Pb layer on the one side and a Cd layer on the other side along the < 111 > direction. Thus, the (111)_PbX_/(111)_CdX_ interface is nearly strain-free.

### Semiconductor Nanomaterial-Based Epitaxial Heterostructures with 1D Seeds/Templates

The synthesis of epitaxial heterostructures with spatially controlled distribution of their chemical composition is very important for constructing multi-functional materials. The use of anisotropic NCs, such as nanorods, nanowires, nanobelts, and branched structures, as platforms to construct heterostructures offers more opportunities for selective deposition of second materials at different locations. In this section, a variety of 0D, 1D, and 2D NCs grown on 1D anisotropic seeds will be introduced.

#### 0D NCs Grown on 1D Seeds

Cadmium chalcogenide NCs are one of the most studied nanomaterials that can form different kinds of anisotropic architectures, such as nanorods and branched structures. The cadmium chalcogenide nanorods or tetrapods consist of 1D sections made of wurtzite structure grown along the *c*-axis. In the wurtzite 1D section, the basal facets are polar and exhibit higher activity as compared to the nonpolar lateral facets. In addition, the wurtzite structure is composed of alternative cadmium and chalcogen atomic layers along the *c*-axis. For example, in a wurtzite CdSe nanorod, the exposed {0001} planes are terminated either by a Cd-terminated (0001) surface or a Se-terminated (000 $\bar{1}$) surface. The Cd atom on the (0001) surface has only one dangling bond, while the Se atom on the (000 $\bar{1}$) surface has three dangling bonds, indicating the different reactivities on these two surfaces. The higher reactivity of the tips in anisotropic NCs offers opportunity for the growth of a second material exclusively at these locations.

The synthesis of nanodumbbells and nanomatchsticks is an interesting example of the site-selective growth [92]. By carefully adjusting the synthetic conditions, matchstick-like or dumbbell-like structures, which consist of PbSe selectively grown on one or both basal facets of CdS or CdSe nanorod, can be obtained. Interestingly, the growth of PbSe on the lateral facets was never observed. The different reactivities among various facets of the wurtzite structure might be related to the different binding energies of the surfactant molecules binding to different facets.

Casavola et al. demonstrated that the facet-dependent chemical reactivity was governed mainly by the selective adhesion of surfactants rather than by the small difference in interfacial strain that results from the lattice mismatch at different locations [93]. In a Co-TiO_2_ system, the Co domains can be selectively decorated either solely at the tips or at multiple locations along the longitudinal sidewalls of TiO_2_ nanorods by adjusting the ratio of different surfactants. The composition and concentration of surfactants in solution are the key parameters in determining the growth mode. Because of their strong affinity to both TiO_2_ and Co surfaces, carboxylic acids, such as 1-octanoic acid (OCAC), are able to form a compact organic shell around the TiO_2_ seeds to inhibit the reaction with the available monomers in solution. Indeed, a high concentration of OCAC in the solution leads to the homogenous nucleation of Co rather than the formation of TiO_2_-Co heterostructure. In contrast, at moderate OCAC/TiO_2_ ratio, matchstick and dumbbell structures are obtained, indicating that the basal facets of TiO_2_ nanorods are less organic-passivated and more chemically accessible than other facets. At a low concentration of OCTA, the ligands covering on the sidewalls of TiO_2_ seeds become poor, so that Co domains start to nucleate at the sidewalls of TiO_2_.

#### 1D NCs Grown on 1D Seeds

##### 1D Core@shell Structure

Compared with the synthesis of onion-like core@shell structures based on 0D seeds, the construction of coaxial core@shell structures with uniform shell thickness based on 1D anisotropic NCs is more challenging. First of all, the synthetic challenge arises from the different surface curvatures among the locations at the edge, tip, and branch regions, which may possess varying chemical reactivities. Since the lengths of these anisotropic NCs are in the range from a few nanometers to hundreds of nanometers, the coaxial growth of epitaxial shells over the extended surface is more challenging. Furthermore, there is an additional requirement to maintain the original shape of the anisotropic seed even though it is not the thermodynamically stable configuration [94].

Analogous to the synthesis of core@shell QDs, Manna et al. reported the growth of CdS@ZnS graded shell on CdSe nanorods by simultaneous injection of the Cd and Zn precursors with a low molar ratio of Cd/Zn [77]. The CdS preferentially nucleates on the surface of CdSe nanorod to reduce the interfacial energy. The larger lattice mismatch between ZnS and CdSe cannot be compensated by the high concentration of Zn in the precursor solution. Interestingly, besides regular shell growth, a tail of ZnS grew straight out of one end of the nanorods after 5–6 monolayers of shell were deposited, providing evidence for the intense strain in the structures. The growth of a ZnS tail at one end of the rod is to relive the strain, which also proves the existence of different reactivities among the surface of nanorod.

##### 1D Linear Heterostructures

The 1D linear heterostructures can be obtained by the extension of the existing 1D seeds, i.e., growth of a second nanorod on the tips of 1D seeds. The mechanism of selective nucleation of 1D nanorod on the tips of 1D seeds is similar to that of the selective deposition of 0D NCs on 1D seeds, except that the 1D directional growth is facilitated in the presence of capping molecules. 1D linear structures of CdTe/CdSe/CdTe can be fabricated by this method using CdSe nanorods as seeds [21, 95, 96]. Experimentally, CdO was heated in a mixture of tetradecylphosphonic acid (TDPA) and TOP to form a starting solution with Cd-TDPA complex as the Cd precursor. The chalcogenide sources were prepared by dissolving Se or Te in TOP. The CdSe nanorod seeds were first formed in the solution by periodically injecting TOP-Se into the reaction solution in every 3 min [95]. Selective growth of CdTe at the tips of CdSe nanorods could be achieved by changing the TOP-Se precursor to TOP-Te. By changing the chalcogenide precursor, the chemical composition of the second phase grown at the tips can be changed. For example, CdSe_*x*_Te_1-*x*_/CdSe/CdSe_*x*_Te_1-*x*_ with continuously variable composition x could be prepared by this method [97].

**Fig. 4:**
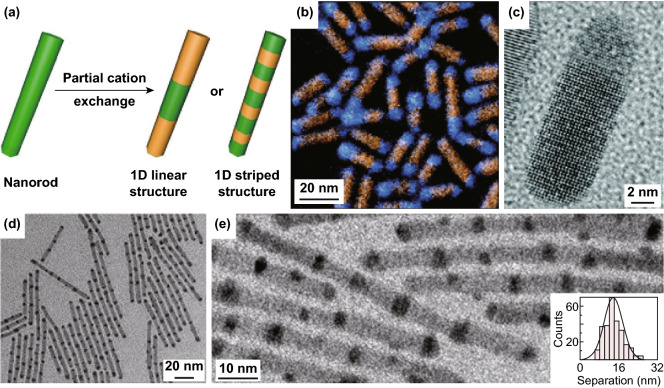
**a** Schematic illustration of the synthesis of 1D linear structure and 1D striped structure by partial cation exchange. **b** Color-composite energy-filtered TEM image of CdS-Cu_2_S nanorods. The orange regions correspond to the Cd energy-filtered mapping, and the blue regions correspond to the Cu mapping. **c** HRTEM image of a CdS-Cu_2_S nanorod. Reproduced with permission from Ref. [98]. Copyright 2009, American Chemical Society. **d, e** TEM images of transformed CdS-Ag_2_S superlattices. Inset in **e**: Histogram of Ag_2_S segment spacing. Reproduced with permission from Ref. [100]. Copyright 2007, Science Publishing Group

The partial cation exchange performed on 1D templates can also result in the formation of 1D linear heterostructures. For example, CdS nanorods can be converted to CdS/Cu_2_S heterostructures by partially replacing the Cd^2+^ ions with Cu^+^ ions [98]. The cation exchange reaction starts preferentially at the tips of CdS nanorods, and the resulting Cu_2_S regions appear to grow along a single crystallographic direction (Fig. 4a). This process results in the formation of Cu_2_S-CdS linear heterostructure (Fig. 4b, c). Since the wurtzite CdS and orthorhombic Cu_2_S share similar anion sublattice, the lattice volume only decreases by 8% when CdS was converted to Cu_2_S, and therefore, the morphology and size of nanorods were well preserved after the cation exchange. Similar structures have been obtained in CdTe/Cu_2-*x*_Te^44^ and CdSe/PbSe [99] systems.

Robinson et al. reported 1D Ag_2_S/CdS striped superstructures by partial cation exchange in CdS nanorods with Ag^+^ ions [100]. At the beginning of the cation exchange, Ag_2_S regions randomly dispersed over the surface of CdS nanorods. This is different from the Cu_2_S-CdS case, in which the cation exchange started preferentially at the tips of CdS nanorods (Fig. 4a) [98]. 1D superlattice with alternating segments of CdS and Ag_2_S along the nanorods was obtained with the progress of cation exchange (Fig. 4d, e).

#### 2D Nanostructures Grown on 1D Seeds

Epitaxial growth of 2D nanosheets or nanoplates on 1D nanowire seeds, referred to as 2D/1D heterostructures, is of great importance to integrate 1D and 2D building blocks into 3D structures with multi-functions. The construction of 2D/1D epitaxial structure is extremely difficult, especially for the 2D building block with atomic thickness, since the epitaxial interface is located at the edge of the growing 2D building block, which requires the perfect match of the crystal structures and the crystal lattices between the two materials.

Bi_2_Te_3_-Te heterostructure consisting of 2D Bi_2_Te_3_ nanoplates grown on 1D Te nanorods is a typical example of 2D/1D epitaxial configurations [101–104]. Wang et al. reported the synthesis of Bi_2_Te_3_-Te heterostructure by a one-pot method. By controlling the reaction kinetics, Te nanorods were produced based on the reaction of TeO_3_^2−^ and Bi^3+^ in the presence of hydrazine. Then, Bi_2_Te_3_ nanoplates grew on the tips and surfaces of these nanorods [101]. The small lattice mismatch and minimization of nucleation energies for Te and Bi_2_Te_3_ were the main reasons for the epitaxial growth of Bi_2_Te_3_ nanoplates on Te nanorods. The lattice mismatch between Te (0001) and Bi_2_Te_3_ (0001) is only 1.62%, allowing the preferential growth of Bi_2_Te_3_ on the (0001) facets located at both ends of Te nanorods. After the extensive epitaxial growth on tips, the Bi_2_Te_3_ started to nucleate on the side surface of Te nanorods. However, due to the significantly larger lattice mismatch on the side surface, the growth of Bi_2_Te_3_ nanoplates on Te nanorod followed the Volmer–Weber model, indicating that Bi_2_Te_3_ nucleates as many isolated islands distributing along the nanorod surface. The further growth of the nuclei led to the formation of Bi_2_Te_3_ nanoplates with (0001) facets perpendicular to the longitudinal direction of Te nanorod.

**Fig. 5:**
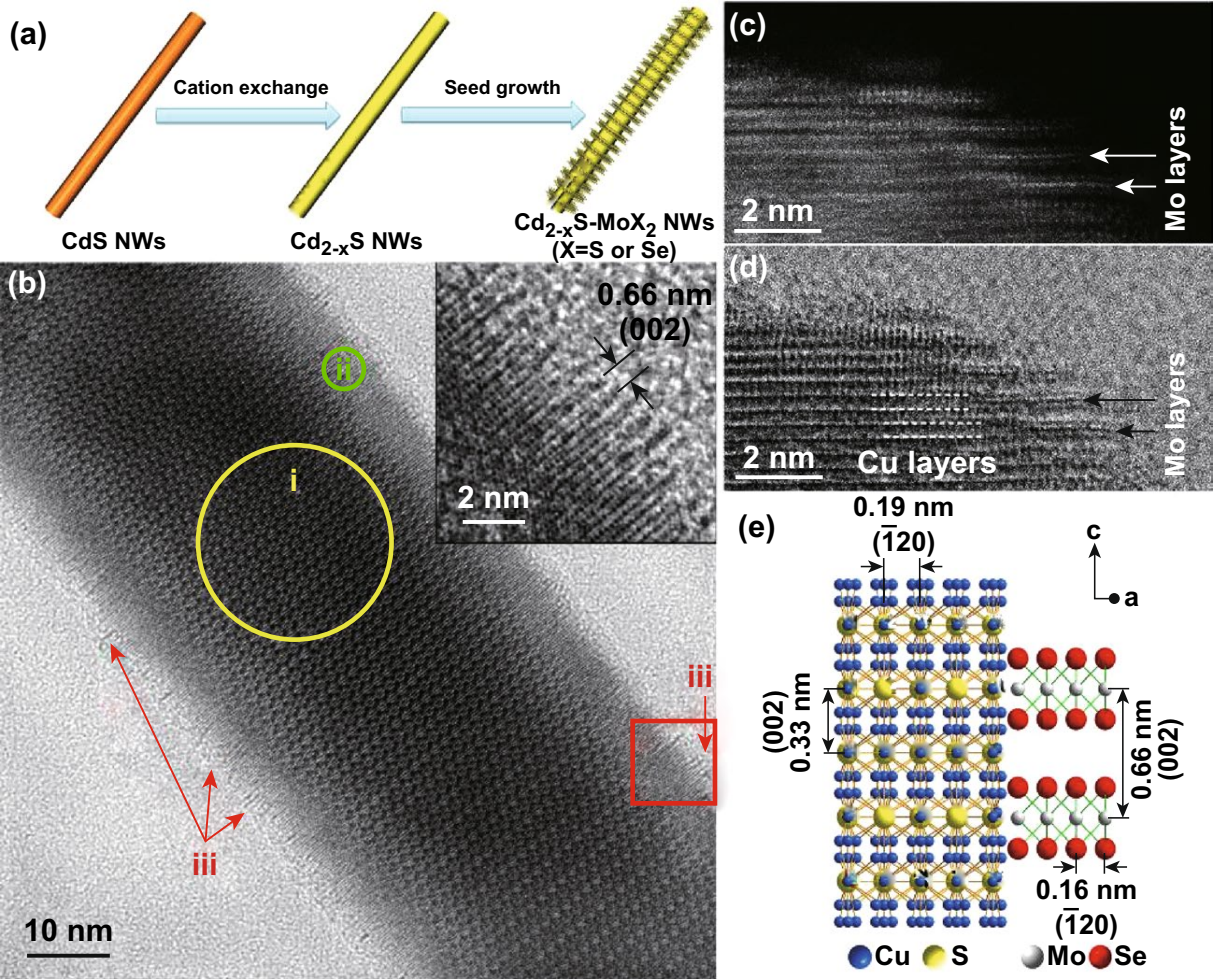
**a** Schematic illustration of the synthesis of Cu_2-*x*_S-MoX_2_ (X = S or Se) epitaxial heterostructure. **b** TEM image of a Cu_2-*x*_S-MoSe_2_ heterostructure. The circles indicate the Cu_1.94_S (component i) and Cu_2_S (component ii). The red arrows indicate the MoSe_2_ (component iii). Inset: HRTEM image of Cu_2-*x*_S-MoSe_2_ taken in the square in (**b**). **c** HAADF-STEM and **d** annular bright-field STEM images of a Cu_2-*x*_S-MoSe_2_ heterostructure taken at the same area. **e** Schematic illustration of the crystal structure of Cu_2-*x*_S-MoSe_2_ heterostructure. Reproduced with permission from Ref. [117]. Copyright 2017, American Chemical Society

Transition metal dichalcogenides (TMDs), e.g., MoS_2_ and MoSe_2_, are layered materials, in which the TMD monolayers stack together by the van der Waals force [105–107]. In the TMD monolayer, a transition metal layer is sandwiched between two chalcogen layers. Due to the special crystal structure, it is extremely challenging to construct TMD-based epitaxial heterostructure with edge contact (i.e., lateral heterostructures) since very few materials have lattice parameters matching the edges of TMDs [24, 108–111]. So far, most of the reported lateral heterostructures are limited to two TMD components, such as MoS_2_-MoSe_2_ and MoS_2_-WS_2_ [112–116]. Recently, our group reported a new type of lateral heterostructure consisting of layered TMD material and another non-layered nanomaterial [117]. The TMD NSs, e.g., MoS_2_ and MoSe_2_, epitaxially grown along the longitudinal direction of 1D Cu_2-*x*_S NWs vertically are realized by using Cu_2-*x*_S NWs as seeds (Fig. 5a, b). In the obtained Cu_2-*x*_S-MoSe_2_ heterostructures, detailed interface structures between the high chalcocite Cu_2_S and MoSe_2_ NSs were revealed by a series of scanning transmission electron microscopy (STEM) characterization (Fig. 5c, d). It was confirmed that the Se layers in MoSe_2_ closely contacted the pure Cu layers in the high chalcocite Cu_2_S. As a result, the well-matched crystal structures between the TMD NSs and Cu_2_S are critical to successfully construct the epitaxial heterostructures (Fig. 5e).

### Semiconductor Nanomaterial-Based Epitaxial Heterostructures with 2D Seeds/Templates

Recently, 2D nanomaterials have attracted tremendous attention due to their superior performance and potential applications in electronics and optoelectronics [118–126]. Two types of 2D nanosheets/nanoplates have been fabricated in recent years. The first type of 2D nanomaterials is made of layered semiconductors which possess 2D crystal structures, such as TMDs. The second type consists of semiconductors with non-layered crystal structures, such as II–VI semiconductors with wurtzite or zinc blende structure [17, 126, 127]. The thickness of both types can be controlled with atomic precision, enabling the 2D nanomaterials with novel physical and chemical properties.

#### 0D NCs Grown on 2D Seeds

**Fig. 6:**
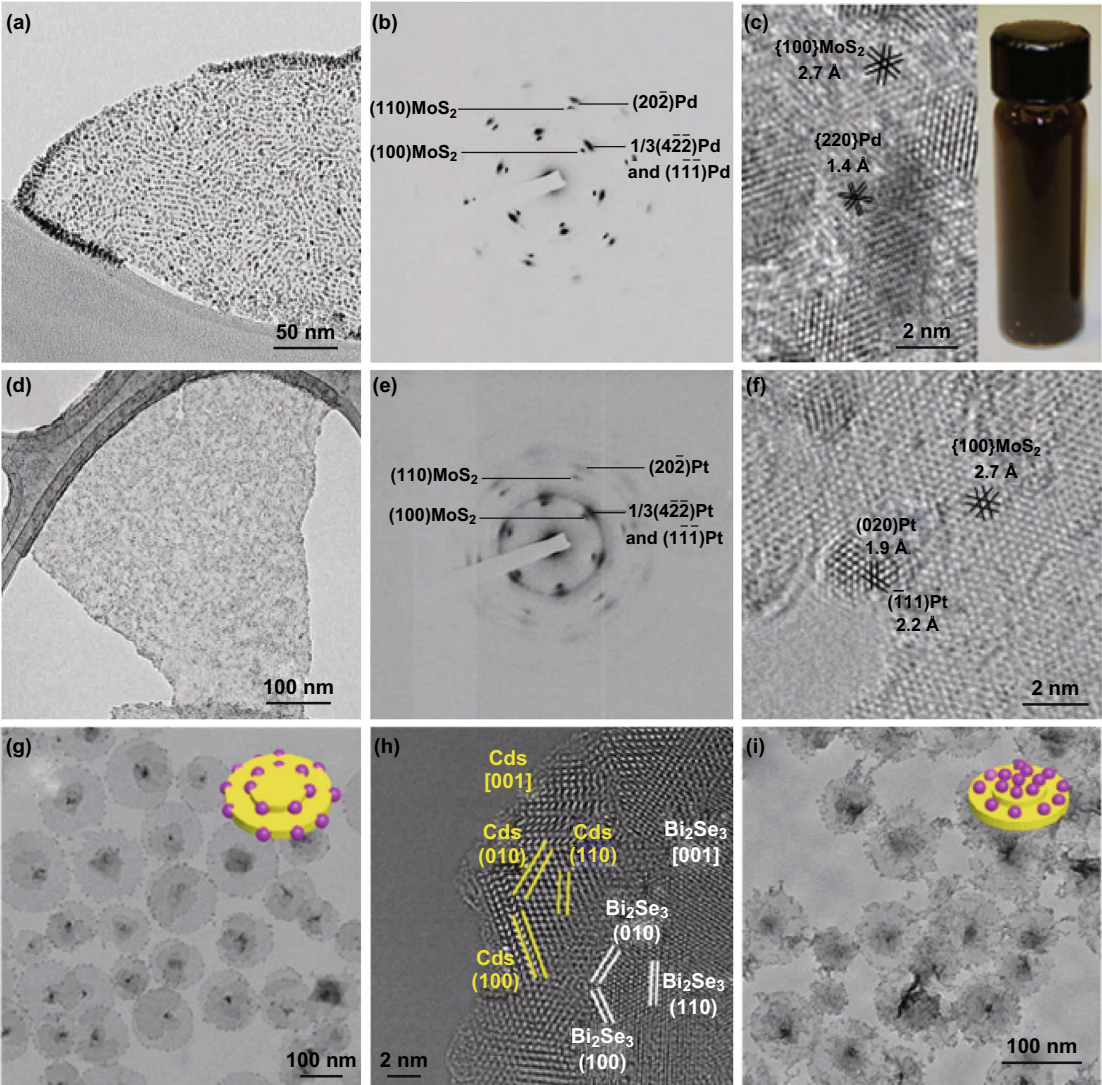
**a** TEM image of Pd NCs grown on an MoS_2_ nanosheet. **b** Selected-area electron diffraction (SAED) pattern of Pd-MoS_2_ epitaxial heterostructures with the electron beam perpendicular to the basal plane of the MoS_2_ nanosheet. **c** HRTEM image of Pd NCs on MoS_2_, showing distinguishable lattice fringes of Pd and MoS_2_. Inset: photograph of the Pd-MoS_2_ solution. **d** TEM image of Pt NCs grown on an MoS_2_ nanosheet. **e** SAED pattern of Pt-MoS_2_ epitaxial heterostructures with the electron beam perpendicular to the basal plane of the MoS_2_ nanosheet. **f** HRTEM image of Pt NCs on MoS_2_ showing distinguishable lattice fringes of Pt and MoS_2_. Reproduced with permission from Ref. [24]. Copyright 2013, Nature Publishing Group. **g** TEM image and schematic illustration of lateral 2D heterostructures consisting of Bi_2_Se_3_ nanoplates and CdS NCs. **h** HRTEM image of the Bi_2_Se_3_-CdS interface, viewed along the [001] axes. **i** TEM image and schematic illustration of basal Bi_2_Se_3_-CdS heterostructures. Reproduced with permission from Ref. [140]. Copyright 2015, American Chemical Society

For 2D nanomaterials with layered crystal structures, the absence of surface dangling bond on the basal plane makes them an excellent platform for construction of van der Waals epitaxial heterostructures which can tolerate very large lattice mismatch [128–130]. However, the weak van der Waals interaction in solution makes it very difficult to synthesize epitaxial heterostructures by wet-chemical methods. Recently, our group demonstrated the epitaxial growth of noble metals NCs, including Pd, Pt, and Ag, on the lithium-intercalated and exfoliated single-layer MoS_2_ NSs in solution phase (Fig. 6a–f) [131]. Two types of epitaxial orientations, i.e., the (111) and (101) orientations, have been found to coexist for metal NCs grown on the MoS_2_ (001) surface. Continuous diffraction rings of {111}, {200}, and {220} of Pt were also identified (Fig. 6e), indicating that not all the Pt NCs exhibit epitaxial growth, probably due to the defects or edges in the MoS_2_ NSs. In addition to noble metals, Schornbaum et al. reported the successful deposition of PbSe QDs on MoS_2_ surface in an epitaxial manner [110]. PbSe QDs with the size of around 5.7 nm were epitaxially grown on MoS_2_ surface with the [200]_PbSe_ lattice planes aligned with the [11–20] _MoS2_ lattice planes.

Recently, lead halide perovskites, such as CH_3_NH_3_PbI_3_, have attracted great attention due to their unique optical properties [132–135]. The growth of perovskite crystals on suitable 2D materials may produce heterostructures with excellent electronic and optical properties [136, 137]. Zhang et al. reported the epitaxial growth of MAPbBr_3_ (MA = CH_3_NH_3_) on MoS_2_ NSs in solution phase [138]. MAPbBr_3_ NCs showed two types of epitaxial relationship with MoS_2_ NSs, i.e., MAPbBr_3_ (200)//MoS_2_ (2 $\bar{1}$ 0) with a 47% lattice mismatch and MAPbBr_3_ (220)//MoS_2_ (100) with a 24% mismatch. Such large symmetry/lattice mismatch was tolerated due to the dangling-bond-free surface of MoS_2_ NSs. Layered 2D NSs without surface dangling bonds are excellent platforms to construct van der Waals epitaxy, which allow the existence of lattice mismatch as large as 50% [139].

The site-selective growth of 0D CdS nanoparticles on either the edge or the basal facet of 2D Bi_2_Se_3_ substrate was demonstrated by Xu and co-workers [140]. In their experiments, by choosing the S precursor with different reactivities, the site-selective growth can be achieved. When sodium diethyldithiocarbamate (DDTC), an inert S precursor, was used, CdS nanoparticles mainly grown laterally on Bi_2_Se_3_ where the (100), (010), and (110) lattice fringes of CdS and Bi_2_Se_3_ were parallel to each other (Fig. 6g, h). In contrast, CdS could be basally grown onto the Bi_2_Se_3_ NSs using more reactive sulfur precursors, such as thiourea and thioacetamide (Fig. 6i). DFT calculations showed that the lateral growth was more preferable compared to the basal growth. When the inert S precursor (i.e., DDTC) was used, CdS preferred to nucleate at the edge, resulting in the lateral growth by a thermodynamically controlled process. However, the kinetically controlled process dominated the synthesis when highly active S precursor (i.e., thioacetamide) was used. The fast decomposition of thioacetamide resulted in a quick nucleation of CdS. The possibility for CdS to deposit onto the lateral or basal plane of Bi_2_Se_3_ was almost the same, due to the presence of more anchoring sites on the large basal plane. This controlled synthesis strategy can be extended to a series of V–VI layered chalcogenides, e.g., Bi_2_Te_3_, Bi_2_Te_2_Se, and Bi_2_TeSe_2_, in which the lateral growth of CdS was achieved.

#### 1D NCs Grown on 2D Seeds

Epitaxial growth of 1D nanostructure on 2D seeds to form 3D architectures is another synthetic challenge. There are only a few reports on the preparation of 3D hierarchical nanostructures from 2D templates [141–143]. The major synthetic obstacle originates from the requirement of the combination of different growth modes and the existence of discontinuous 1D/2D interfaces. Moreover, it is still difficult to control the nucleation and growth sites of secondary materials on the anisotropic 2D seeds to form specific architectures.

Trizio et al. reported the growth of CdSe nanorods/nanoplates on the basal facets of Cu_3_P nanoplates in a mixture of trioctylphosphine oxide (TOPO), hexylphosphonic acid (HPA), and octadecylphosphonic acid (ODPA) [142]. The preferential growth of CdSe nanorods at the edge of the basal facets of the Cu_3_P plates was realized at 300 °C. The selective growth was most likely driven by the higher activity of the Cu_3_P edges, on which more active sites existed for the nucleation of CdSe. The small lattice mismatch between the (0002) facets of CdSe and Cu_3_P facilitated the vertical growth of CdSe nanorods on Cu_3_P nanoplates. Interestingly, sandwich-like heterostructures were obtained at high reaction temperature (380 °C) in the presence of a large amount of ODPA. These results indicated that the capping molecules (HPA and ODPA) may change the surface chemical state of Cu_3_P and control the nucleation of CdSe on Cu_3_P. However, more experiments are required to reveal the detailed growth mechanism.

**Fig. 7:**
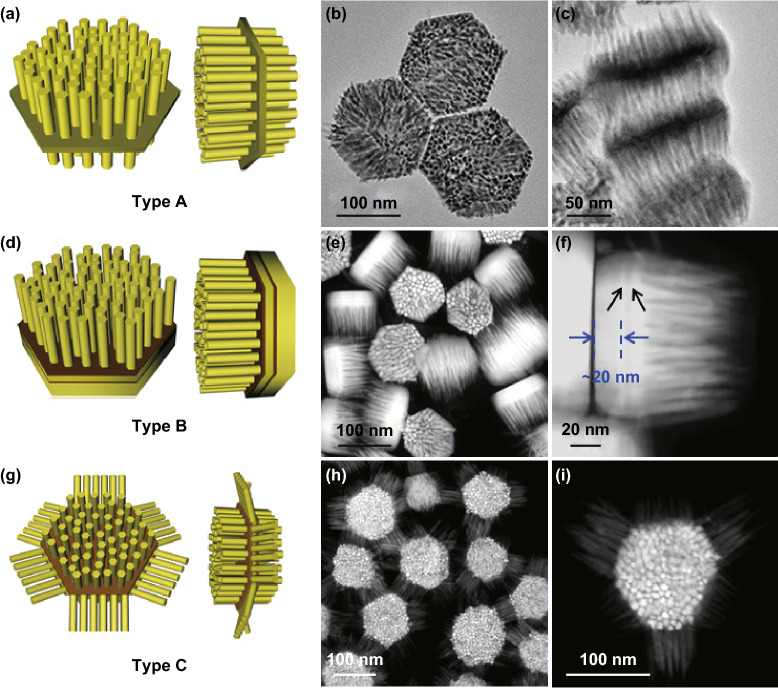
**a** Schematic illustration of type A nanostructure. TEM images of CdS-type A nanostructure lying flat **b** and standing **c** on the TEM grids. **d** Schematic illustration of type B nanostructure. **e** Dark-field STEM image of CdS-type B nanostructures. **f** Dark-field STEM image of a typical CdS-type B nanostructure standing on the TEM grid. Two bright strips, indicated by black arrows, can be clearly observed in the nanostructures. **g** Schematic illustration of type C nanostructure. **h, i** Dark-field STEM images of CdS-type C nanostructures. Reproduced with permission from Ref. [141]. Copyright 2016, Nature Publishing Group

Our group reported a seeded growth approach to construct three types of epitaxial 1D/2D heterostructures, in which II–VI semiconductor (CdS or CdSe) nanorod arrays can be selectively grown on different facets of hexagonal-shaped nanoplates, i.e., the two basal facets of the nanoplate (type A, Fig. 7a–c), one basal facet (type B, Fig. 7d–f), or the two basal facets and six side facets (type C, Fig. 7g–i) [141]. The 1D/2D heterostructures were prepared by injecting the S (or Se) precursor with different types of seeds into a reaction solution containing Cd precursor and capping molecular (i.e., ODPA, HPA, TOP, and TOPO). The seed-engineering is essential for the construction of these hieratical 1D/2D structures. Three types of seeds originated from hexagonal CuS nanoplates are prepared. Type A structure was synthesized by using wurtzite CdS nanoplates (seed A) as seed, which exposed basal (001) and (00 $\bar{1}$) facets. CdS or CdSe nanorods selectively grow on both basal facets of the wurtzite CdS seed along its < 001 > direction (Fig. 7b, c). The mechanism for such directional growth is similar to that used for the preparation of dot-in-rod structures discussed in Sect. 3.1.2. Type B structure was synthesized by using a sandwich seed consisting of two hexagonal Cu_2-*x*_S_*y*_Se_1-*y*_ layers and a cubic Cu_2-*z*_S core (*h*-Cu_2-*x*_S_*y*_Se_1-*y*_/Cu_2-*z*_S/*h*-Cu_2-*x*_S_*y*_Se_1-*y*_, seed B). Interestingly, asymmetric growth was observed in type B structure, in which nanorods were only grown on one basal facet of seed B, but on the other basal facet of seed B, a uniform CdS coating layer was observed (Fig. 7e, f). Similar to the CdSe nanorod grown on Cu_3_P [134], the capping molecular (ODPA, HPA, TOP) as well as the lattice mismatch induced by different chemical compositions may have a great effect on the growth mode. Unfortunately, the detailed mechanism is still unclear. Type C structure was synthesized by using a sandwich seed consisting of two cubic Cu_2-*x*_S_*y*_Se_1-*y*_ layers and a cubic Cu_2-*z*_S core (*c*-Cu_2-*x*_S_*y*_Se_1-*y*_/Cu_2-*z*_S/*c*-Cu_2-*x*_S_*y*_Se_1-*y*_, seed C). Impressively, CdS nanorod arrays grown on six side facets and two basal facets of the seed C were obtained (Fig. 7h, i). During the synthesis of type C structures, the sandwich-like seed C underwent a fast cation exchange reaction in the presence of excessive Cd^2+^ ions. The crystal structure after cation exchange can be ascribed to the zinc blende structure with well-developed exposed {111} facets. Therefore, the wurtzite CdS nanorods epitaxially grew on the eight {111} facets, i.e., two basal and six side facets.

#### 2D NCs Grown on 2D Seeds

##### 2D van der Waals Heterostructures

Stacking 2D layered materials on top of each other to form van der Waals heterostructures has emerged and led to the observation of numerous exciting physical phenomena [109, 144]. Lots of van der Waals heterostructures have been constructed by the CVD growth or physical epitaxy. However, direct synthesis of such kind of heterostructures in solutions is rarely reported. Recently, Wang and co-workers reported the realization of vertical metal–semiconductor heterostructures composed of Sn_0.5_W_0.5_S_2_ and SnS_2_ in solution phase by a one-pot hydrothermal method [145]. SnS_2_ nanoplates and Sn_0.17_WO_3_ nanorods were formed at the beginning of the hydrothermal process. As the reaction proceeded, the Sn_0.17_WO_3_ decomposed and Sn_0.5_W_0.5_S_2_ NSs started to epitaxially deposit on the surface of the SnS_2_ nanoplates. The combination of semiconducting SnS_2_ and metallic 1T Sn_0.5_W_0.5_S_2_ led to the formation of ohmic-like contact at the Sn_0.5_W_0.5_S_2_/SnS_2_ heterointerface. Thus, the obtained heterostructures showed rapid transport of charge carriers and allowed for the fabrication of fast photodetectors.

##### 2D Core@shell Structures

**Fig. 8:**
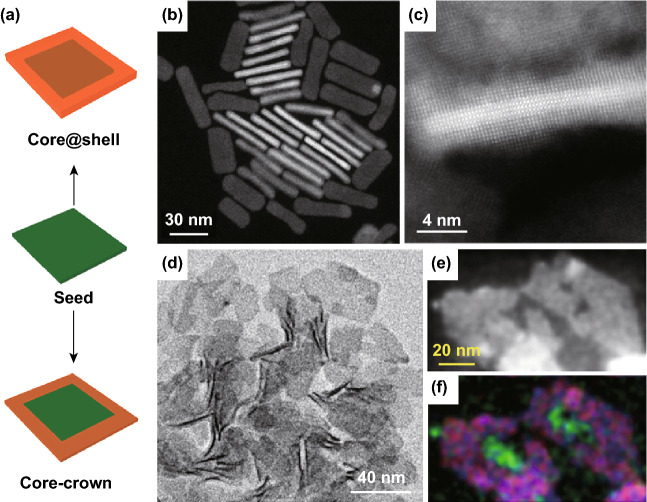
**a** Schematic illustration of the synthesis of core@shell and core-crown structures from 2D seeds. **b** HAADF image of annealed CdSe@CdS core@shell nanoplates with very smooth surfaces. **c** High-resolution HAADF image of a CdSe@CdS core@shell nanoplate on the side exhibiting the core@shell structure and five Cd planes of the initial CdSe nanoplate. Reproduced with permission from Ref. [146]. Copyright 2012, American Chemical Society. **d** TEM image of CdSe-CdS core-crown heterostructures. **e** HAADF-STEM image and **f** color-coded STEM EDS map of CdSe-CdS core-crown heterostructures. The red, green, and blue colors represent to S, Se, and Cd, respectively. Reproduced with permission from Ref. [151]. Copyright 2013, American Chemical Society

Two methods have been developed to prepare epitaxial 2D core@shell nanoplates, such as CdSe@CdS. Mahler et al. developed a layer-by-layer deposition at room temperature, through which the layer number of the deposited shells can be precisely controlled [146]. For example, to deposit a CdS monolayer on CdSe nanoplate, a bis-trimethylsilylated (TMS_2_S) anion layer, acted as the S precursor, was first coated on zinc blende CdSe surface by ligand exchange. Then, the nanoplate was washed twice to remove the excess of TMS_2_S. Such washing step also created a reactive and S-rich surface on CdSe by breaking the remaining TMS-S bonds. Then, a solution of Cd acetate was added to induce the monolayer growth at room temperature (Fig. 8a–c). The synthetic approach is very general and can be extended to other types of shell materials, as long as the reactivities of the core surface and the precursor are high [147–150].

Although the layer-by-layer method is very useful to finely control the shell thickness and chemical composition, this protocol is very time-consuming, and the NCs need to be carefully washed between each deposition process to avoid the secondary nucleation. To simplify the experiment, a one-pot method was developed based on the in situ generation of H_2_S by the reaction of thioacetamide with octylamine [146]. In the experiment, a mixture of thioacetamide (TAA) and octylamine was first added into the CdSe solution to initiate the deposition of a sulfur layer. Then, a cadmium source was added, and the reaction was allowed to proceed for a few hours to obtain CdSe@CdS core@shell structure. However, compared with the layer-by-layer method, the shell deposition using the one-pot method was not uniform. Some nanoplates showed distorted shape and others had edges with higher contrast than their center, indicating the thickness inhomogeneity.

##### 2D Core-Crown Structures

Another type of 2D/2D heterostructure is the core-crown structure, in which the second material is grown laterally to the original 2D seed, while the thickness remains unchanged after reaction (Fig. 8a). For example, Prudnikau et al. reported the synthesis of CdSe-CdS core-crown structures (Fig. 8d–f) [151]. The formation of these structures was realized by using Cd ethylhexanoate and S in octadecene as precursors for the lateral growth of CdS in the presence of short-chain carboxylate ligands. In contrast, long-chain ligands may block the epitaxial growth at the edges of CdSe seed, resulting in the formation of core@shell structure. Compared with the CdSe-CdS core@shell structure which shows a large (~ 100 nm) red shift of absorption and PL bands, the CdSe-CdS core-crown structure shows almost no red shift of the exciton transition after the CdS growth. The absence of red shift is due to the unchanged thickness after the growth of CdS.

## Properties and Applications of Semiconductor Nanomaterial-Based Epitaxial Heterostructures

Until now, a wide spectrum of semiconductor nanomaterial-based epitaxial heterostructures with varying architectures and compositions has been prepared by wet-chemical methods. These nanomaterials with unique optical and electric properties have shown many potential applications, including light-emitting devices (LEDs) [152–154], solar cell [155–157], photodetectors [110], bio-imaging/sensing [158], and catalysis [33, 35]. In this section, we will briefly introduce the unique advantages of epitaxial heterostructures prepared via wet-chemical methods in some promising applications.

### Light-Emitting Devices

**Fig. 9:**
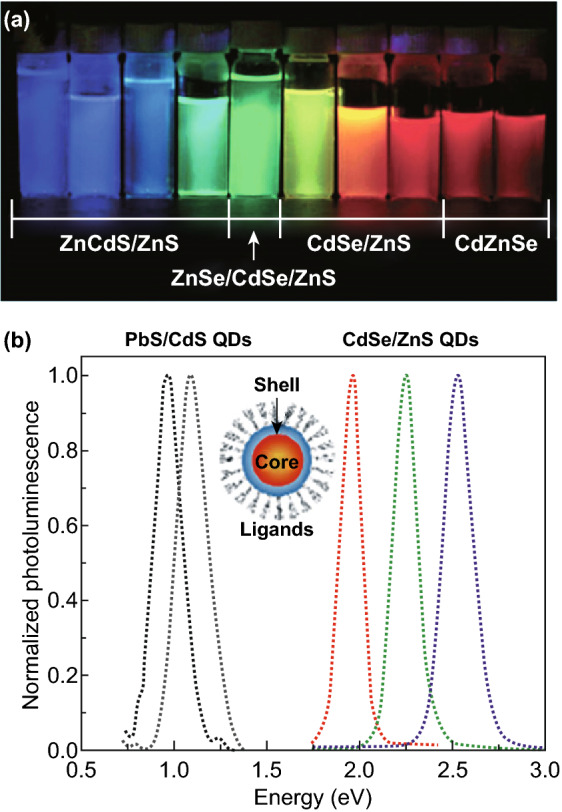
**a** Photograph of PL of core@shell QDs with different compositions. In this photograph, QD solutions are excited by a UV lamp with emission centered at a wavelength of 365 nm. Reproduced with permission from Ref. [161]. Copyright 2009, American Chemical Society. **b** PL spectra of CdSe@ZnS and PbS@CdS core@shell QDs showing the possibility for narrowband emission across the visible and near-IR wavelength regions. Inset: schematic illustration of a core@shell QD. Reproduced with permission from Ref. [160]. Copyright 2010, Nano Reviews

Colloidal QDs with type I core@shell structure have been widely used for light-emitting applications [159–161]. The great advantage of QDs for LED applications arises from their tunable bandgap, high quantum efficiency (QE), and solution processability. The ability to precisely control the size and shape of QDs allows researchers to fine-tune the bandgap of QDs due to the quantum size effect. Together with the various chemical compositions and stoichiometries, systematic and precise spectral tunability of efficient emission can be achieved (Fig. 9a). For example, CdSe-based core@shell QDs are used for the visible-wavelength LEDs, and PbS-based heterostructure is the material of choice for near-infrared devices, as shown in Fig. 9b.

The core@shell configuration of QDs with epitaxial shell greatly enhances the QE and photostability [162]. The epitaxial shell can passivate the surface non-radiative recombination sites of the core. The surface dangling bonds, acting as trap states for the charge carriers and thereby reducing the QE, are reduced by the epitaxial shell [163]. In addition, since the shell physically separates the optically active core from the surrounding medium, the sensitivity of optical properties is reduced and the photostability of the QDs is enhanced. For example, the PL quantum yield of phase-pure zinc blende CdSe@CdS core@shell QDs with ten-monolayer CdS shell can achieve above 90%, which was almost one order of magnitude greater than that of native CdSe cores [159]. Based on these high-quality CdSe@CdS QDs, LED with excellent performance and reproducibility, color-saturated emission, and high efficiency up to 20.5% can be fabricated. Besides the core@shell structures based on 0D seeds, core@shell nanoplates [153], dot-in-rod [164], and double-heterostructure nanorods [154, 165–167] have also been used to fabricate LEDs.

### Photovoltaic Device

The basic processes in a photovoltaic device include light absorption, subsequent photoexcitation, charge separation, and charge collection [168, 169]. The charge carrier separation process is a critical step for efficient solar energy conversion. Semiconductor epitaxial heterostructures with type II staggered band offset are capable of efficiently separating photoexcited charges, a property which is highly desirable for photovoltaics. In addition, the most efficient configuration for charge transfer across heterointerface is the high-quality epitaxial interfaces. The epitaxy between different semiconductor components promotes direct electronic communication. For example, ultrafast separation of photoexcited carriers has been demonstrated in CdSe-CdTe type II heterostructures with different geometries. CdS@CdTe core@shell QDs [170], quantum rods [171], nanoplates [172], and terapods [85] exhibit sub-*ps* charge separation rates with slow recombination. However, the ability of colloidal heterostructures to improve photovoltaic performance is challenged by some undesirable aspects, such as surface oxidation, charge traps, and inefficient charge transport. The charge transport through a film made of colloidal heterostructures is quite inefficient due to the existence of surface traps and hopping barriers [155]. Thus, charge transport through a film of colloidal heterostructures should be avoided when these materials are used in photovoltaics. McDaniel et al. reported photovoltaics that utilized CdSe/CdTe 1D linear heterostructure as an extremely thin absorber between the electron and hole transporting layers [173]. The photovoltaics incorporated with CdSe/CdTe heterostructures showed a short-circuit current and conversion efficiency up to three times as high as other devices made from single-component counterparts of the heterostructures.

Another strategy to efficiently separate photoexcited charges is to directly epitaxially grow electron donor materials on acceptor materials. Acharya et al. reported the direct epitaxial growth of PbS QDs (donor) on a TiO_2_ (acceptor) film on indium tin oxide (ITO) substrate using hot-injection method [174]. The photovoltaic performance of PbS/TiO_2_ epitaxial heterostructure was evaluated by incorporating PbS/TiO_2_ films into the prototype solar cells, in which the maximum power conversion efficiency was 1.2%.

Perovskite solar cells based on organic–inorganic halide perovskites have attracted much research interest due to their high efficiency and low cost. High-quality perovskite films with large grain size and preferential orientation are very important to achieve high-performance perovskite solar cells [175]. Recently, Tang et al. reported a solution-phase van der Waals epitaxy growth of MAPbI_3_ perovskite films on MoS_2_ flakes [135]. The in-plane coupling between the perovskite and MoS_2_ crystal lattices leads to larger grain size, lower trap density, and preferential growth orientation along (110) of the perovskite films. Therefore, in perovskite solar cells, the power conversion efficiency is substantially enhanced for 15% due to the increased crystallinity of the perovskite layer and the improved hole extraction and transfer rate at the interface.

### Catalysis

**Fig. 10:**
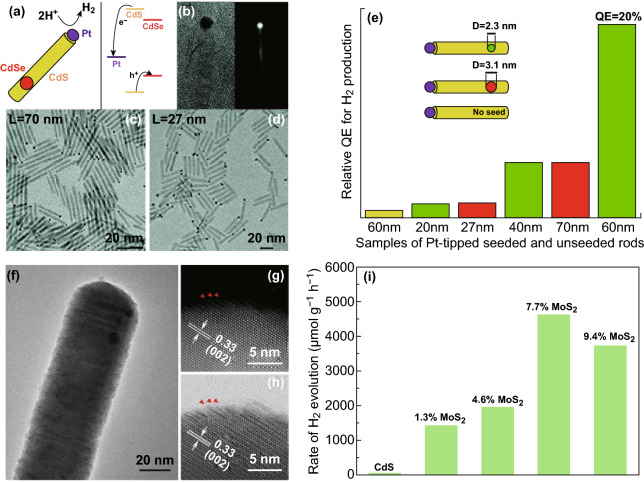
**a** Schematic illustration of the multi-component heterostructure and energy band diagram of a Pt-tipped CdS rod with an embedded CdSe seed. **b** The Pt tip is clearly seen in the HRTEM and HAADF-STEM images. TEM images of Pt-tipped seeded rods with the lengths of **c** 27 nm and **d** 70 nm on average. **e** Relative quantum efficiency for hydrogen production, obtained from Pt-tipped unseeded CdS rods (yellow), and five different samples of Pt-tipped seeded rods with seed diameters of 3.1 (red) or 2.3 nm (green). Reproduced with permission from Ref. [176]. Copyright 2010, American Chemical Society. **f** TEM image of a CdS-MoS_2_ heterostructure. **g** HAADF-STEM and **h** ABF-STEM images of the tip area of a CdS-MoS_2_ heterostructure. The red triangles indicate the position of MoS_2_ NSs. **i** Comparison of H_2_ production activities using CdS NWs and CdS-MoS_2_ heterostructures with different loading amounts of MoS_2_ NSs as catalysts. Reproduced with permission from Ref. [117]. Copyright 2017, American Chemical Society. (Color figure online)

The semiconductor-based epitaxial heterostructures have also been used as photocatalysts for photocatalytic hydrogen evolution (HER) and photodegradation of methylene blue. Amirav et al. reported the design of a multi-component photocatalyst composed of Pt-tipped CdSe-CdS dot-in-rod heterostructure for HER (Fig. 10a–d) [176]. The unique advantage of this heterostructure lies in its ability to efficiently separate the photoexcited charges. In such structure, holes are three-dimensionally confined to the CdSe cores, whereas the electrons are transferred to the Pt tips. Consequently, the electrons are separated from holes over three different components (Fig. 10a). By tuning the size of CdSe core and the length of CdS rod, significant improvement of the activity for hydrogen production as compared to that of CdS-Pt was achieved (Fig. 10e). However, the problem in such structure lies in the non-epitaxial relationship between CdS nanorod and Pt particle, which may give rise to dense interfacial defects, dissipating the excitation energy [177].

Our group evaluated the HER activity of CdS-MoS_2_ epitaxial heterostructures synthesized by a facial cation exchange using Cu_2-*x*_S-MoS_2_ epitaxial heterostructures as templates [117]. After cation exchange, the obtained CdS-MoS_2_ heterostructures remained their original architectures (Fig. 10f–h). The vertical growth of MoS_2_ along the longitudinal direction of 1D CdS nanowires in an epitaxial manner was obtained. The CdS-MoS_2_ heterostructures with 7.7 wt % loading of MoS_2_ nanosheets exhibited much enhanced performance in the photocatalytic HER with a H_2_ production rate up to 4,647 μmol h^−1^ g^−1^, which was about 58 times as high as that catalyzed by pure CdS NWs (Fig. 10i). The significant improvement in HER activity may result from the epitaxial configuration between MoS_2_ and CdS. The direct contact between CdS and MoS_2_ through the edge of MoS_2_ may promote the charge transfer between these two components.

The epitaxial heterostructures also exhibit enhanced catalytic activities toward photodegradation of organic dyes, compared to the non-epitaxial counterparts. Wang et al. reported the synthesis of CdS/α-Fe_2_O_3_ heterostructure with α-Fe_2_O_3_ nanoparticles epitaxially grown on the CdS nanowire [35]. Compared to the non-epitaxial CdS/Fe_3_O_4_ composites, the CdS/α-Fe_2_O_3_ epitaxial heterostructure showed higher photocatalytic activity toward the photodegradation of methylene blue. The enhanced activity of the CdS/α-Fe_2_O_3_ may be contributed to the faster charge separation in the CdS/α-Fe_2_O_3_ than that in the CdS/Fe_3_O_4_.

### Thermoelectric Device

Thermoelectric devices, such as noiseless cooling and power generation devices, have attracted great attention due to its ability to directly convert between thermal and electrical energies [178]. However, the performance of thermoelectric devices is still far from satisfactory due to the low figure of merit (ZT). The formation of the nearly perfect epitaxial interface in epitaxial heterostructure, which could suppress the thermal transport and greatly maintain the electronic properties, is desirable for thermoelectric applications. The previous studies have shown that some thermoelectric materials, such as Bi_2_Te_3_/Sb_2_Te_3_ (ZT ~ 2.4) superlattice film, possess high thermoelectric performance because of the improved phonon scattering at the interfaces and grain boundaries. However, the extremely expensive and time-consuming fabrication process, e.g., molecular beam epitaxy, is required [179]. Lin et al. reported a gram-scale solution-phase growth of PbSe/Bi_2_Se_3_ epitaxial heterostructure by using 2D Bi_2_Se_3_ nanoplate as the template [13]. The thermoelectric device made from PbSe/Bi_2_Se_3_ showed a thermoelectric figure-of-merit ZT three times higher than that of the pristine Bi_2_Se_3_ nanoplates at 575 K. This significant enhancement can be largely attributed to the increased Seebeck coefficient and ultralow thermal conductivity of the epitaxial heterostructures, resulting from the efficient phonon scattering at the PbSe and Bi_2_Se_3_ epitaxial interface.

Theoretical studies have predicted that the further enhancement of thermoelectric figure of merit could be achieved in 1D heterostructures, such as superlattice nanowire, by taking the advantages of both the quantum confinement to enhance the power factor and the phonon scattering at heterointerface to the lower thermal conductivity [180, 181]. Experimentally, Zhang et al. reported the synthesis of Te-Bi_2_Te_3_ barbell heterostructure by selectively converting the Te nanowire into Bi_2_Te_3_ [181]. The density of hexagonal Bi_2_Te_3_ plates on the Te nanowire can be controlled. The electrical conductivity and the Seebeck coefficient of Te-Bi_2_Te_3_ heterostructure are much higher than those of the Te nanowire. As a result, the ZT value of the Te-Bi_2_Te_3_ heterostructure is more than two orders greater than the pure Te nanowire. The significant improvement of the thermoelectric performance is due to the enhanced phonon scattering at the nanowire heterostructure surface and interface.

## Conclusion and Outlook

This review focuses on the progress that has been made over the past decades in the field of wet-chemical synthesis of semiconductor-based epitaxial heterostructures and their applications. Seed-mediated solution-phase epitaxial growth and post-synthesis cation exchange method are especially highlighted. Epitaxial heterostructures with various architectures and different compositions synthesized using 0D, 1D, and 2D NCs as seeds/templates are summarized. The growth mechanisms for each type of structures are discussed. The unique advantages originated from the epitaxial configuration have endowed these heterostructures with great potential in a number of applications ranging from optoelectronics, catalysis to thermoelectrics.

Although great progress has been made, some challenges still remain in the synthesis of semiconductor-based epitaxial heterostructures. First, further studies are required to gain more insights into the growth mechanisms for the formation of different kinds of epitaxial heterostructures. Designing elaborate epitaxial heterostructures with precise control of structures and compositions requires more understanding on the growth mechanism under various conditions. Lots of factors, including the match of crystal structures, surface energies, capping molecules, and kinetic and thermodynamic aspects, have been proposed to explain the epitaxial growth of various heterostructures. However, there is still lack of the general principle to guide the design of synthetic routes for epitaxial heterostructures in order to meet specific functional requirements.

Second, from the structure point of view, designing epitaxial heterostructure with mixed dimensionality is essential for exploring their potential applications. Materials with different dimensionalities possess their own intrinsic advantages and disadvantages. The construction of heterostructures with mixed dimensionalities could combine the advantages and mitigate the drawbacks of the single components. For example, in the dot-in-rod heterostructure, the band edge emission is related to the 0D core, while some advantages, such as linearly polarized emission, suppression of Auger non-radiative recombination, and large absorbance cross sections, are inherited from the 1D shell [182]. Transition metal dichalcogenides (TMDs), such as MoS_2_ and MoSe_2_, are 2D layered materials with anisotropic properties. For example, the in-plane conductivity of MoSe_2_ is ~ 1500 times as high as the cross-plane conductivity [183]. Thus, the construction of mixed-dimensional heterostructures consisting of 2D TMDs and other non-layered semiconducting materials with interface located at the edges of TMDs may possess tremendous potential applications due to the anisotropic properties of TMDs. However, until now the construction of epitaxial heterostructure with mixed dimensionality is still a great challenge, especially for heterostructures consisting of 1D and 2D units [117, 141].

Third, further efforts are also required from the application point of view. Although semiconductor epitaxial heterostructures have shown great potential in many applications, such as LED and phototransistors, the relationship between epitaxy and certain properties remains unclear. For example, significant blinking (fluorescence intermittency) of QDs limits their applications as single-photon light sources and biolabels for tracking single biomolecules [184]. Generally, a thick inorganic shell (> 5 nm) coated on the QDs is required to suppress the blinking [184, 185]. However, recent results showed that the blinking could be efficiently suppressed by a thin high-quality epitaxial shell (~ 0.7 nm), but the detailed mechanism is still unclear [20]. Except some applications, such as catalysis, few attention has been paid to how the epitaxial growth affects the application. Therefore, more efforts should be devoted to this research field for preparing various semiconductor-based epitaxial heterostructures and exploring their promising applications.
